# First molecular phylogenetic insights into the evolution of *Eriocaulon* (Eriocaulaceae, Poales)

**DOI:** 10.1007/s10265-019-01129-3

**Published:** 2019-08-05

**Authors:** Isabel Larridon, Norio Tanaka, Yuxi Liang, Sylvia M. Phillips, Anders S. Barfod, Seong-Hyun Cho, Stephan W. Gale, Richard W. Jobson, Young-Dong Kim, Jie Li, A. Muthama Muasya, John A. N. Parnell, Amornrat Prajaksood, Kohtaroh Shutoh, Phetlasy Souladeth, Shuichiro Tagane, Nobuyuki Tanaka, Okihito Yano, Attila Mesterházy, Mark F. Newman, Yu Ito

**Affiliations:** 1 Royal Botanic Gardens, Kew, Richmond, Surrey TW9 3AE UK; 2 Department of Botany, National Museum of Nature and Science, 4-1-1 Amakubo, Tsukuba, Ibaraki 305-0005 Japan; 3 Department of Bioscience, Aarhus University, Ny Munkegade 114, 8000 Aarhus C, Denmark; 4 Multidisciplinary Genome Institute, Hallym University, Chuncheon, 24252 Korea; 5 Kadoorie Farm and Botanic Garden, Lam Kam Road, Tai Po, New Territories, Hong Kong, SAR China; 6 National Herbarium of New South Wales, Royal Botanic Gardens and Domain Trust, Mrs Macquaries Road, Sydney, NSW 2000 Australia; 7 Xishuangbanna Tropical Botanical Garden, Plant Phylogenetics and Conservation Group, Chinese Academy of Sciences, Kunming, 650223 China; 8 Department of Biological Sciences, University of Cape Town, Bolus Herbarium, Private Bag X3, Rondebosch, 7701 South Africa; 9 Herbarium, Botany Department, Trinity College Dublin, Dublin 2, Ireland; 10 Department of Biology, Faculty of Science, Khon Kaen University, Khon Kaen, 40002 Thailand; 11 The Hokkaido University Museum, Hokkaido University, Kita 10, Nishi 8, Kita-ku, Sapporo, Hokkaido 060-0810 Japan; 12 National University of Laos, Dongdok Campus, Xaythany District, Vientiane Capital, Lao PDR; 13 The Kagoshima University Museum, Kagoshima University, 1-21-30 Korimoto, Kagoshima, 890-0065 Japan; 14 Department of Biosphere-Geosphere Science, Faculty of Biosphere-Geosphere Science, Okayama University of Science, Ridai-cho 1-1, Kita-ku, Okayama, Okayama 700-0005 Japan; 15 Directory of Hortobágy National Park, Sumen utca 2, Debrecen, 4024 Hungary; 16 Royal Botanic Garden Edinburgh, 20A Inverleith Row, Edinburgh, Scotland EH3 5LR UK; 17 Faculty of Pharmaceutical Sciences, Setsunan University, Osaka, Japan

**Keywords:** Aquatic plants, Eriocaulaceae, Evolution, Molecular phylogenetics, Monocots

## Abstract

*Eriocaulon* is a genus of c. 470 aquatic and wetland species of the monocot plant family Eriocaulaceae. It is widely distributed in Africa, Asia and America, with centres of species richness in the tropics. Most species of *Eriocaulon* grow in wetlands although some inhabit shallow rivers and streams with an apparent adaptive morphology of elongated submerged stems. In a previous molecular phylogenetic hypothesis, *Eriocaulon* was recovered as sister of the African endemic genus *Mesanthemum*. Several regional infrageneric classifications have been proposed for *Eriocaulon*. This study aims to critically assess the existing infrageneric classifications through phylogenetic reconstruction of infrageneric relationships, based on DNA sequence data of four chloroplast markers and one nuclear marker. There is little congruence between our molecular results and previous morphology-based infrageneric classifications. However, some similarities can be found, including Fyson’s sect. *Leucantherae* and Zhang’s sect. *Apoda*. Further phylogenetic studies, particularly focusing on less well sampled regions such as the Neotropics, will help provide a more global overview of the relationships in *Eriocaulon* and may enable suggesting the first global infrageneric classification.

**Electronic supplementary material:**

The online version of this article (10.1007/s10265-019-01129-3) contains supplementary material, which is available to authorized users.

## Introduction

*Eriocaulon* L., commonly known as pipeworts, is a cosmopolitan genus of ephemeral and perennial aquatic and wetland plants of the Eriocaulaceae family (Poales). The genus includes c. 470 species (WCSP 2019) and is most species-rich in Asia (c. 220 species), the Americas (c. 122 species) and Africa (c. 111 species), with its centres of diversity in the tropics (Stützel 1998). Species of *Eriocaulon* primarily grow in seasonal or permanent wetlands while some inhabit shallow rivers and streams with an apparent adaptive morphology of elongated submerged stems. Two subfamilies are recognised in Eriocaulaceae, i.e. Eriocauloideae with diplostemonous flowers and glandular petals, and Paepalanthoideae with isostemonous flowers and eglandular petals (Giulietti et al. 2012; Ruhland 1903). Together with the African genus *Mesanthemum* Körn., which was recently revised by Liang et al. (2019), *Eriocaulon* is placed in subfamily Eriocauloideae. Subfamily Paepalanthoideae is largely restricted to the Americas.

Despite the ecological importance of *Eriocaulon* as a species-rich genus of wetland plants, no attempts have been made to reconstruct a molecular phylogeny for the genus. Only a few *Eriocaulon* species have been included in the sampling of family level studies (e.g. de Andrade et al. 2010; Giulietti et al. 2012). A molecular phylogenetic study including a broad sampling covering much of the taxonomic, morphological and geographic variation within the genus is needed to assess whether the infrageneric taxa suggested in the existing regional infrageneric classifications of *Eriocaulon* circumscribe monophyletic groups. It is a first step in providing insights into the evolution of the genus and to enable establishing a new infrageneric classification for the whole genus in the future.

Several regional infrageneric classifications of the species of *Eriocaulon* have been proposed. Mueller (1859) established two sections to accommodate the then known Australian species of *Eriocaulon*, i.e. sect. *Dimorphogyne* F. Muell. to accommodate *E*. *heterogynum* F. Muell. and sect. *Eriocaulon* L. was established as autonym to place the remaining six Australian species (Table S1).

Fyson (1919, 1921, 1922) established an infrageneric classification for the Indian species of *Eriocaulon*, placing 51 species in eight named sections (Table S1). Later, Ansari and Balakrishnan (1994, 2009) proposed an infrageneric classification of twelve numbered sections (I–XII) for the Indian species of *Eriocaulon* (Table S1). There is little overlap between these two classification systems for India, although both Fyson (1919, 1921, 1922) and Ansari and Balakrishnan (1994, 2009) place *E. alpestre* Hook.f. & Thomson ex Körn. in a monotypic section (i.e. sect. *Comato*-*Sepalae* Fyson and sect. I; Table S1). Also, species of sect. *Hirsutae* Fyson appear to be mostly placed in sect. II and III by Ansari and Balakrishnan (1994, 2009), while species of sect. *Leucantherae* Fyson appear to be placed in sect. XII.

Ma (1991) classified the Chinese species of *Eriocaulon* into subgen. *Trimeranthus* Nakai (27 species) and subgen. *Eriocaulon* sensu Nakai (monotypic: *E*. *decemflorum* Maxim.). He further divided subgen. *Trimeranthus* into three sections: sect. *Macrocaulon* Ruhl. (16 species); sect. *Leucocephala* Nakai (three species); and sect. *Spathopeplus* Nakai (eight species). *Eriocaulon* sect. *Macrocaulon* comprised ser. *Tmetopsis* Ruhl. (11 species) and ser. *Leiantha* W.L.Ma (4 species), while sect. *Spathopeplus* consisted of ser. *Miqueliana* Satake (2 species), ser. *Robustiora* W.L.Ma (4 species), and ser. *Manshanensia* W.L.Ma (2 species) (Ma 1991) (Table S1). Later Ma (1997) added sect. *Macrocaulon* ser. *Disepala* Satake to retrieve *E*. *merrillii* Ruhl. and *E*. *sclerophyllum* W.L.Ma from ser. *Leiantha* (Table S1). The numbers of species classified were 32 in Ma (1997) compared to Ma (1991) who accepted 28 species. Ma et al. (2000) accepted 35 species in China and rejected all infrageneric classifications.

Zhang (1999) proposed an infrageneric classification which placed 71 East Asian species in two subgenera and 10 sections (Table S1), recognising some of the sections used by Fyson (1919, 1921, 1922) and Ma (1991, 1997) together with some additional sections. There is little overlap between the classifications of Zhang (1999) and Ansari and Balakrishnan (1994, 2009). However, *E*. *hamiltonianum* Mart. and *E*. *truncatum* Buch.-Ham. ex Mart. are grouped in sect. VII in Ansari and Balakrishnan (1994, 2009) and sect. *Disepala* in Zhang (1999).

None of the published regional infrageneric classifications have yet been scrutinised using molecular phylogenetic data. A molecular study by de Andrade et al. (2010) on Eriocaulaceae included just five species of *Eriocaulon* while the study of Giulietti et al. (2012) included just four species. Of the species sequenced in these studies, *E*. *cinereum* R.Br. is the only one that has been included in the published infrageneric classifications. The objectives of this study are to: (1) construct a molecular phylogeny of *Eriocaulon*, and (2) critically assess the existing regional infrageneric classifications of *Eriocaulon*.

## Materials and methods

### Taxon sampling

**Fig. 1:**
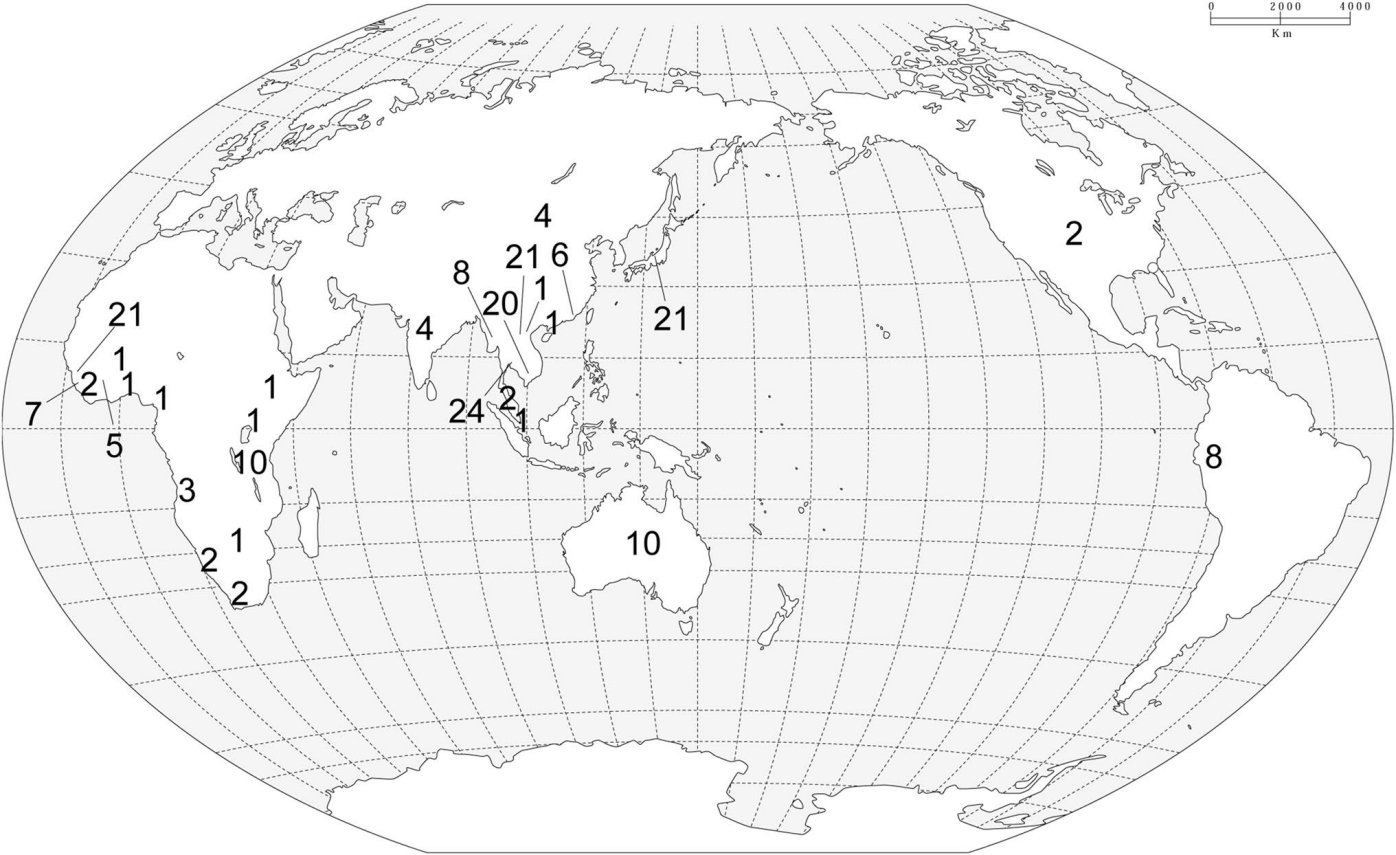
Map of sampling localities of *Eriocaulon* species included in this study indicating the number of accessions sampled at each location

Samples of *Eriocaulon* were collected in the field or obtained from herbarium specimens (Table S2). The following regional treatments were used for specimen identifications because no comprehensive global revision has been published: Cook (1996) and Ansari and Balakrishnan (2009) for India; Prajaksood et al. (2017) for Thailand; Ma et al. (2000) for China; Miyamoto (2015) for Japan; Bentham (1878) and Leach (1992, 2000, 2017) for Australia; Cook (2004) for southern Africa; Meikle (1968) for west tropical Africa; Phillips (1998, 2010, 2011) for east and southern tropical Africa. Cook (1996), Ma (1991, 1997) and Ma et al. (2000) were referred to identify Indo-Burma specimens. The recently described species *E*. *petraeum* S.M.Phillips & Burgt and *E*. *sulanum* S.M.Phillips & Burgt (Phillips et al. 2012) were sampled. Our sampling included 199 accessions (116 from Asia; 59 from Africa; 14 from America; ten from Australia) from 79 ingroup species representing 16.8% of species diversity of the genus *Eriocaulon* (Fig. 1; Table S2). *Xyris* Gronov. of Xyridaceae and *Mesanthemum*, *Syngonanthus* Ruhl. and *Tonina* Aubl. of Eriocaulaceae, were chosen as outgroup taxa following de Andrade et al. (2010).

### DNA extraction, amplification and sequencing

Total genomic DNA was extracted from silica gel-dried leaf tissues using the CTAB method described in Ito et al. (2010). Four regions of chloroplast DNA (ptDNA), i.e. *matK*, *rbcL*, *rpoB* and *rpoC1* were PCR amplified with the following primers: matK-390F (Cuénoud et al. 2002) and matK-1520R (Whitten et al. 2000) for *matK*; rbcL-F1F (Wolf et al. 1994) and rbcL-1379R (Little and Barrington 2003) for *rbcL*; “2f” and “4r” for *rpoB* (Royal Botanic Gardens, Kew); and “1f” and “3r” for *rpoC1* (Royal Botanic Gardens, Kew). The PCR amplification was conducted using TaKaRa Ex Taq polymerase (TaKaRa Bio, Shiga, Japan), and PCR cycling conditions were 94 °C for 60 s; then 30 cycles of 94 °C for 45 s, 52 °C for 30 s, 72 °C for 60 s; and finally, 72 °C for 5 min.

The PHYC gene (a distinct member of the phytochrome gene family) was selected as a nuclear marker, based on its phylogenetic utility as a single or low copy nuclear locus (Mathews and Donoghue 1999; Samuel et al. 2005). Fragments of a part of exon 1 of PHYC were amplified by PCR using Comm_PHYC_P1F (Hertweck et al. 2015) and the newly designed AlisPHYC-1R (5′-GCATCCATTTCMACATCYTCCCA). The PCR cycling conditions were 94 °C for 90 s; then 35 cycles of 94 °C for 45 s, 60 °C for 30 s, 72 °C for 90 s; and finally, 72 °C for 10 min. The fragments obtained were digested with ExoSAP-IT and directly sequenced.

The PCR products were cleaned using ExoSAP-IT (GE Healthcare, Piscataway, NJ, USA) purification, and then amplified using ABI PRISM Big Dye Terminator v.3.1 (Applied Biosystems, Foster City, CA, USA) using the same primers as those used for the PCR amplifications. DNA sequencing was performed with an ABI PRISM 377 DNA sequencer (Applied Biosystems). Automatic base-calling was checked by eye in Genetyx-Win v.3 (Software Development Co., Tokyo, Japan). All sequences generated in the present study have been submitted to the DNA Data Bank of Japan (DDBJ), which is linked to GenBank, and their accession numbers and voucher specimen information are presented in Table S2.

### Molecular phylogenetic analyses

Sequences were aligned using MAFFT v.7.058 (Katoh and Standley 2013) and then inspected manually. Analyses were independently performed for ptDNA (*matK*, *rbcL*, *rpoB*, *rpoC1*) and PHYC datasets respectively to identify possible incongruences between different genomic regions. All 199 ingroup and the 13 outgroup accessions were represented in the ptDNA dataset, while 55 ingroup and five outgroup accessions were represented in the PHYC dataset. The ptDNA dataset consisted of concatenated gene alignments with 145 or 68% of accessions represented for *matK*, 197 or 93% for *rbcL*, 122 or 58% for *rpoB* and 41 or 19% for *rpoC1*.

Phylogenies were reconstructed using maximum parsimony (MP), maximum likelihood (ML), and Bayesian inference (BI; Yang and Rannala 1997). In the MP analysis in PAUP* v.4.0b10 (Swofford 2002), a heuristic search was performed with 100 random addition replicates with tree-bisection-reconnection (TBR) branch swapping, with the MulTrees option in effect. The MaxTrees option was set at 100,000. Bootstrap analyses (Felsenstein 1985) were performed using 1,000 replicates with TBR branch swapping and simple addition sequences. The MaxTrees option was set at 1,000 to avoid entrapment in local optima.

For the ML analysis, the RAxML BlackBox online server (https://www.raxml-ng.vital-it.ch/) was used, which supports GTR-based models of nucleotide substitution (Stamatakis 2006). The maximum likelihood search option was used to find the best-scoring tree after bootstrapping. The gamma model of rate heterogeneity was selected. Statistical support for branches was calculated by rapid bootstrap analyses of 100 replicates (Stamatakis et al. 2008).

BI analyses were conducted with MrBayes v.3.2.2 (Ronquist and Huelsenbeck 2003; Ronquist et al. 2012) run on the CIPRES portal (Miller et al. 2010) after the best models had been determined in MrModeltest v.3.7 (Nylander 2002); these models were GTR + I + G and GTR + G for ptDNA and PHYC datasets, respectively. Analyses were run for 6,335,000 and 1,500,000 generations for ptDNA and PHYC datasets, respectively, until the average standard deviation of split frequencies dropped below 0.01, sampling every 1,000 generations and discarding the first 25% as burn-in. The convergence and effective sampling sizes (ESS) of all parameters were checked in Tracer v.1.6 (Rambaut et al. 2014). All trees were visualized using FigTree v.1.3.1 (Rambaut 2009). Support values are provided at the nodes [MP bootstrap support (BS), ML BS, BI posterior probability (PP)].

### Molecular dating

A species tree was used to conduct a molecular dating analysis. A multispecies coalescent method (Heled and Drummond 2009) implemented in BEAST v.1.7.2 (Drummond et al. 2006; Drummond and Rambaut 2007) was performed to reconstruct a species tree. *BEAST was run using a multilocus dataset (ptDNA and PHYC) utilising all 212 ingroup and outgroup samples assigned to the 84 operational taxonomic units (OTUs) that were retrieved as clades in the phylogenetic analyses above. For the purposes of this analysis, species resolved as non-monophyletic or that contained multiple lineages are represented multiple times in the resulting tree (i.e. *E*. *cinereum* R.Br., *E*. *latifolium* Sm., *E*. *nepalense* J.D.Prescott ex Bong., *E*. *plumale* N.E.Br. and *E*. *setaceum* L.).

A relaxed molecular clock as implemented in BEAST v.2.4.4 (Drummond et al. 2006) was used and run on the CIPRES portal (Miller et al. 2010). Uncorrelated lognormal distributed substitution rates for each branch were used. The tree was rooted by constraining Eriocaulaceae. Previous divergence time estimates between Eriocaulaceae and Xyridaceae of 105 mya (million years ago) provided by Janssen and Bremer (2004), Bouchenak-Khelladi et al. (2014) and Hertweck et al. (2015) were used as a calibration point. These dates were set as a mean age with stdev = 0.1 and a normal distribution. A Yule speciation process was used as tree prior. The default settings of BEAUti v.2.4.4 were used for the other parameters. Two runs of ten million generations of the MCMC chains were run, sampling every 1,000 generations. Convergence of the stationary distribution was checked by visual inspection of plotted posterior estimates using Tracer v.1.6 (Rambaut et al. 2014). After discarding the first 1,000 trees as burn-in, the samples were summarised in the maximum clade credibility tree using TreeAnnotator v.1.6.1 (Drummond and Rambaut 2007) with a posterior probability (PP) limit of 0.5 and summarizing mean node heights. The results were visualised using FigTree v.1.3.1 (Rambaut 2009).

## Results

### Molecular phylogeny

**Fig. 2:**
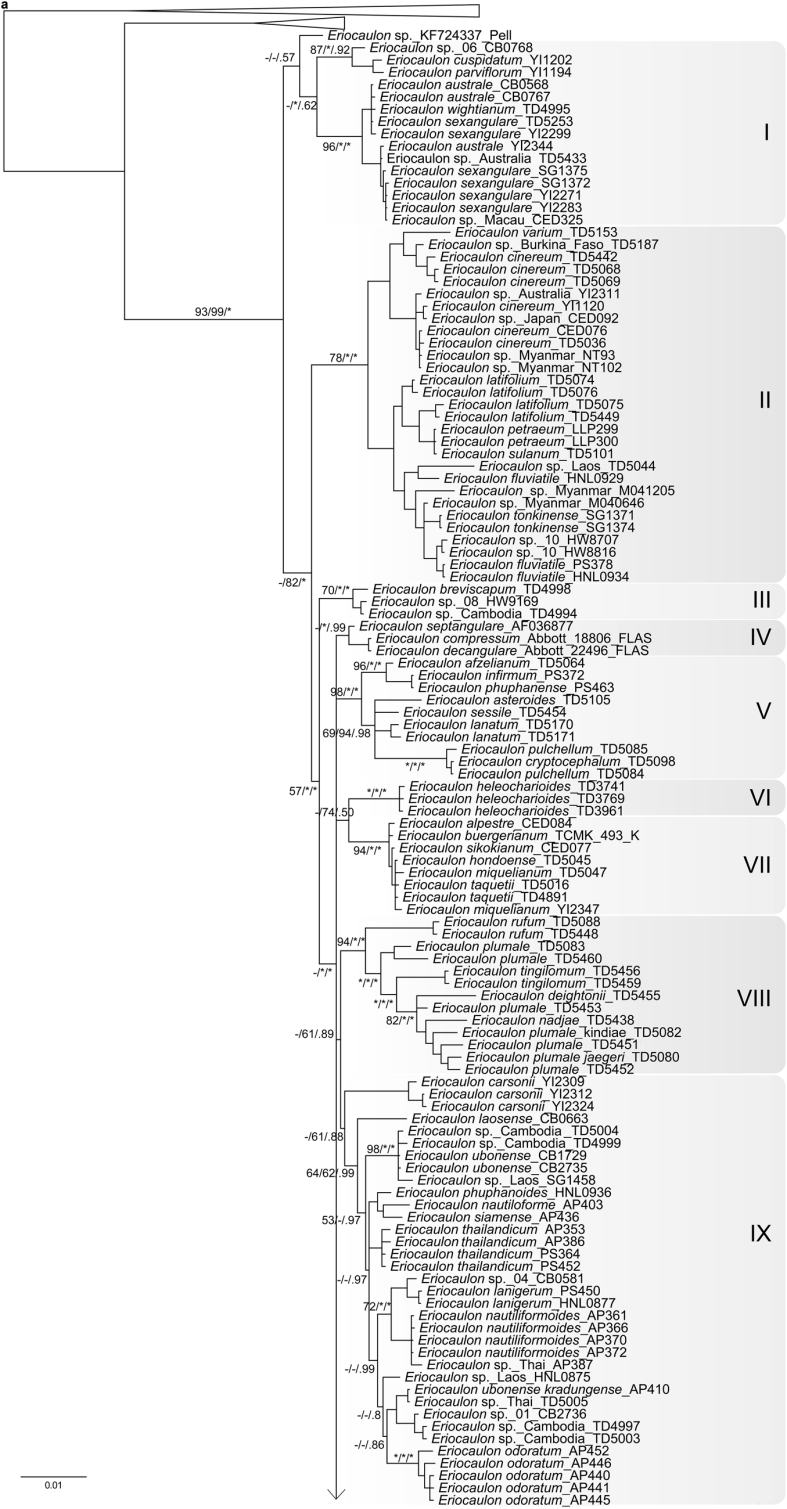

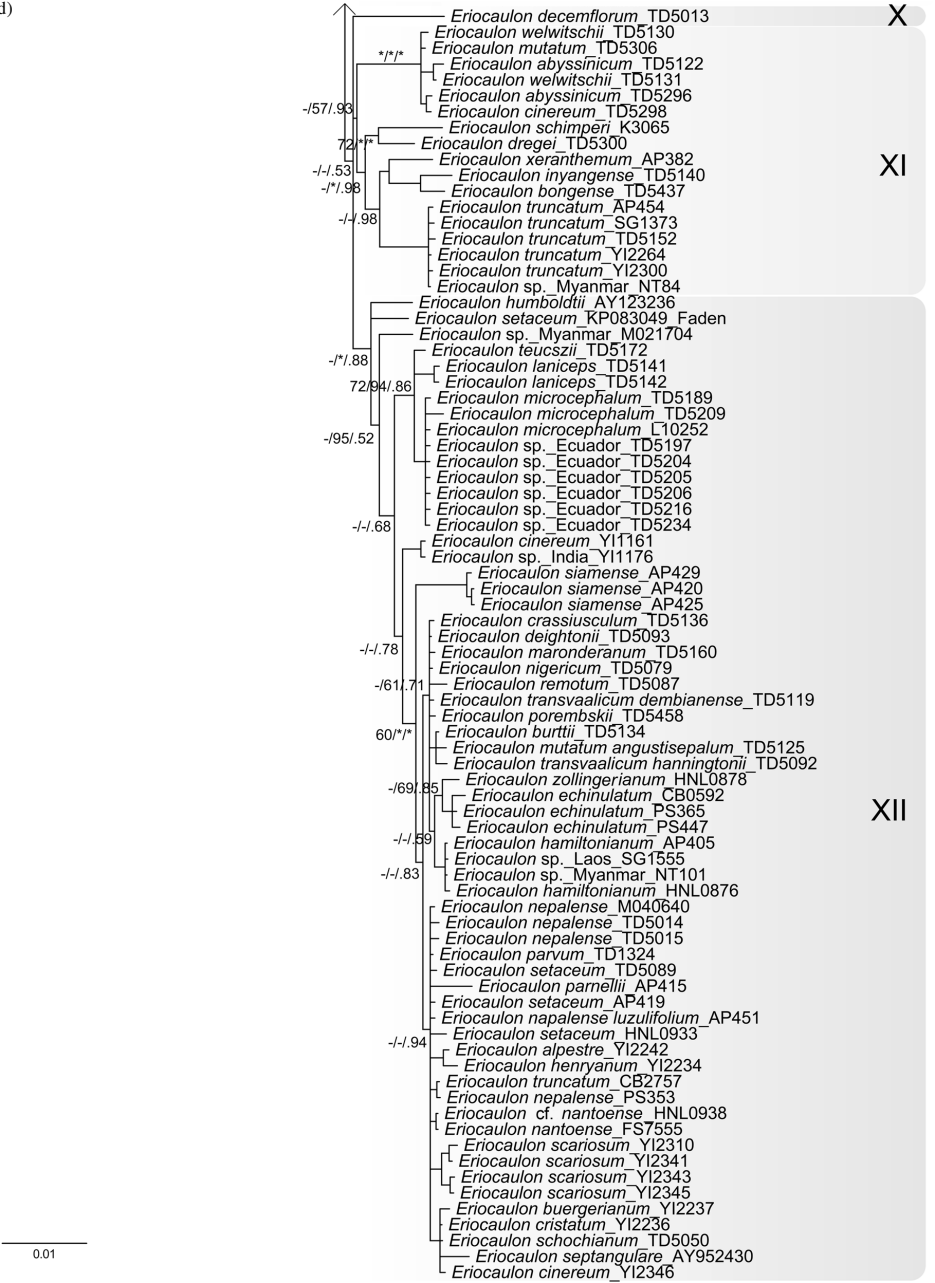

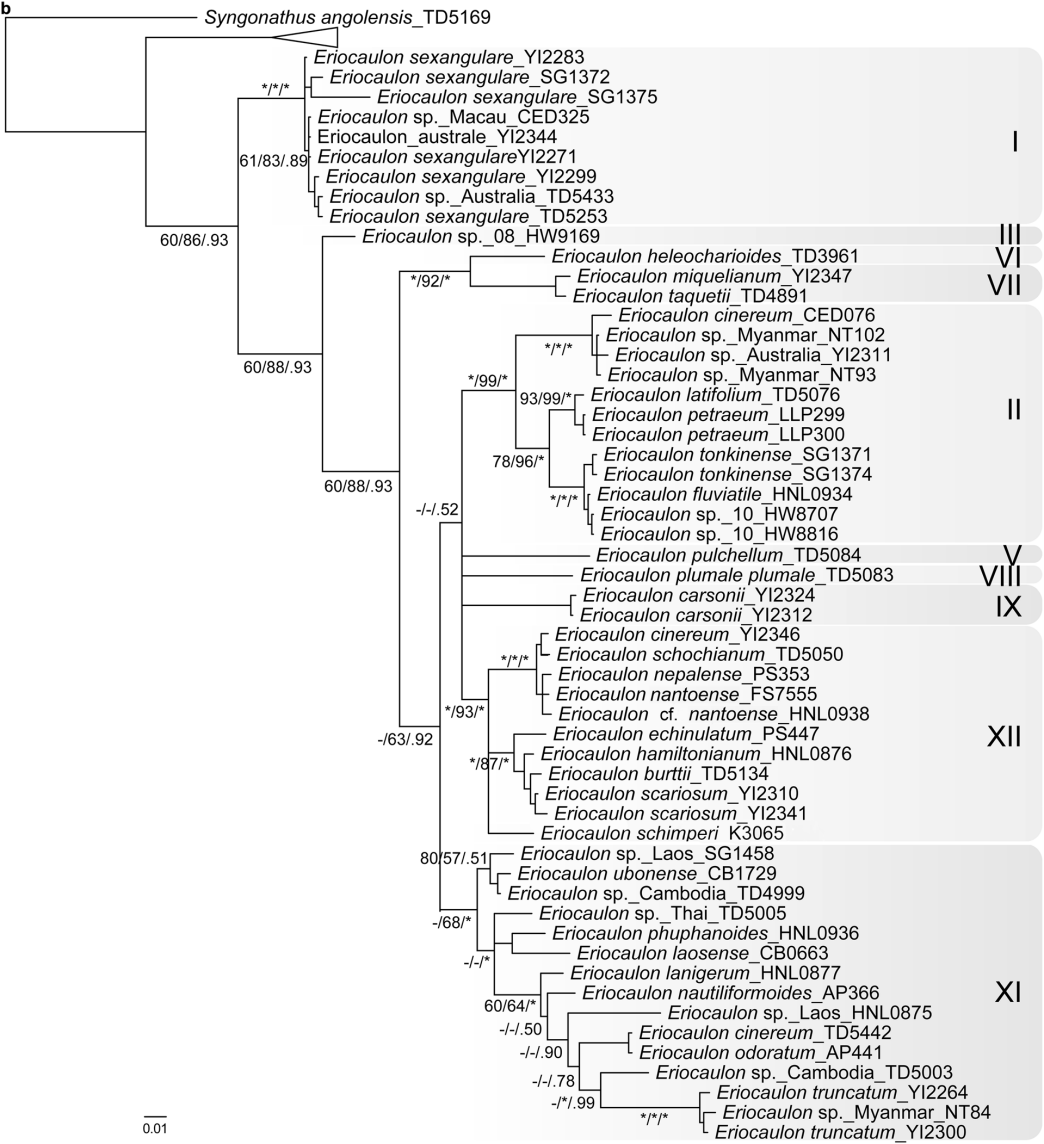
MrBayes trees of *Eriocaulon* based on: **a** concatenated plastid DNA and **b** nuclear PHYC datasets. Samples collected in this study are associated with the specified vouchers. Branch lengths are proportional to the number of substitutions per site as measured by the scale bar. Values above the branches represent the maximum parsimony and maximum likelihood bootstrap support values (MP BS/ML BS), and Bayesian posterior probabilities (PP). BS < 70% and PP < 0.9 are indicated by hyphens while those of ≥ 90% and ≥ 0.95 are shown as asterisks. Well-supported clades are highlighted by gray rectangles

The ptDNA dataset for four genes included 4,445 aligned characters, of which 889 were parsimony informative. Analysis of this dataset yielded the imposed limit of 100,000 MP trees (tree length = 2,590 steps; consistency index = 0.64; retention index = 0.88). The strict-consensus MP tree, the RAxML tree, and the MrBayes BI 50% consensus tree showed no incongruent phylogenetic relationships; thus only the BI tree is presented here (Fig. 2a). *Eriocaulon* is broadly divided into two lineages: clade I and clades II–XII. The clade II is resolved as sister to clades III–XI. Clade III is resolved as sister to clades IV–XII. The relationships among clades IV–XII are less well resolved, except the weakly supported clades VIII–IX, yet each clade is highly supported. Singleton X is differentiated from clades XI–XII.

The PHYC dataset included 979 aligned characters, of which 324 were parsimony informative. Analysis of this dataset yielded the imposed limit of 100,000 MP trees (tree length = 1,181 steps; consistency index = 0.53; retention index = 0.81). The strict-consensus MP tree, the RAxML tree and the MrBayes BI 50% consensus tree showed no incongruent phylogenetic relationships; thus, only the BI tree is presented here (Fig. 2b). The labelling of PHYC tree follows the ptDNA tree. *Eriocaulon* is broadly divided into two lineages: Clade I and clades/singletons II–III, V–IX and XI–XII. Singleton III is resolved as sister to clades/singletons II–XII. Singleton VI and clade VII are retrieved as sister lineages, as are clades/singletons II, V, VIII–IX and XI–XII. The relationships in the latter group are less resolved. Clade II is strongly supported. Clade XII plus Eriocaulon_schimperi_K3065 which belongs to clade XI in the ptDNA analysis are strongly supported as a natural group. Members of clade XI except Eriocaulon_schimperi_K3065 are retrieved as a clade.

### Molecular dating

**Fig. 3:**
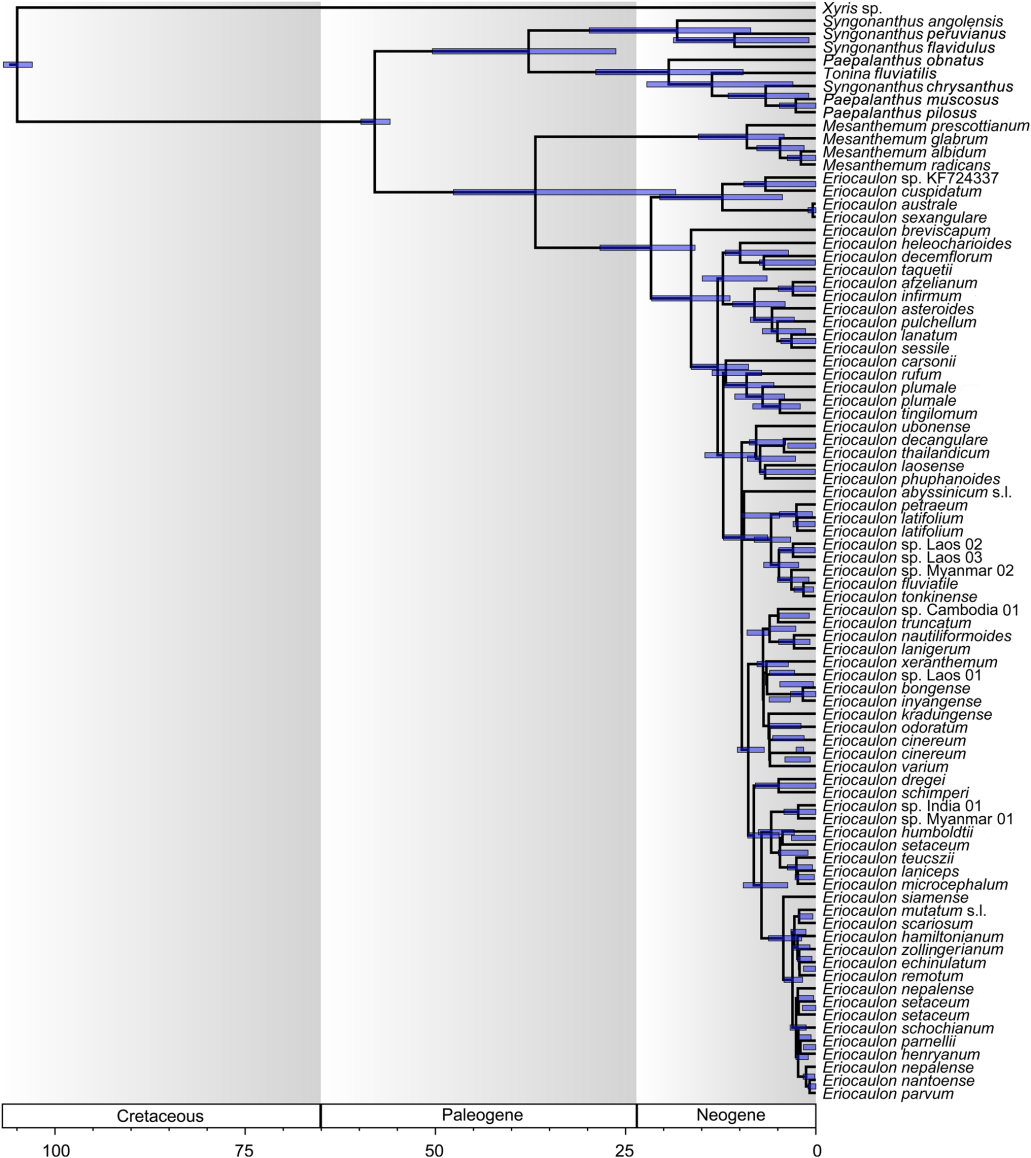
BEAST maximum clade credibility tree for *Eriocaulon* obtained from plastid DNA (*mat*K, *rbc*L, *rpo*B and *rpo*C1) and nuclear PHYC sequence data. Clade depth and bars indicate mean nodal ages (mya) and 95% highest posterior density intervals

The divergence time for each clade was estimated using the calibration point between Eriocaulaceae and Xyridaceae of 105 mya provided by Janssen and Bremer (2004), Bouchenak-Khelladi et al. (2014) and Hertweck et al. (2015). The most recent common ancestor (MRCA) of the Eriocaulaceae family was estimated as early Paleogene with the Eriocauloideae MRCA as mid-Paleogene. The approximate divergence time for the MRCA of *Eriocaulon* was estimated as late Paleogene to early Neogene (21.66 mya; 95% HDP = 15.88–28.36 mya) (Fig. 3). Most of the species diversity of *Eriocaulon* appears to have originated in the the last 10 mya (Fig. 3).

## Discussion

### Phylogeny and systematics of *Eriocaulon*

We reconstructed the phylogenetic history of *Eriocaulon* using both ptDNA and PHYC datasets with the aim of assessing the existing regional infrageneric classifications (Table S1). Although our taxon sampling is not sufficiently comprehensive to cover Mueller’s (1859) sectional classification for Australian species of *Eriocaulon*, selected species listed in the infrageneric classifications proposed by Fyson (1919, 1921, 1922), Ma (1991, 1997), Ansari and Balakrishnan (1994, 2009) and Zhang (1999) were sampled (Table S1, S2). Here, using the ptDNA tree (Fig. 2a), we discuss whether and how the results support these infrageneric classifications of as well as the previous molecular phylogeny of de Andrade et al. (2010). There is little congruence between our molecular results and previous morphology-based infrageneric classifications. However, some similarities can be found, as detailed below.

In de Andrade et al. (2010) ptDNA tree, *Eriocaulon cinereum* was retrieved as sister to the other four species including *E*. *decangulare* L. Our ptDNA tree recovered a similar topology in which *E*. *cinereum* of clade II branches off before *E*. *decangulare* of clade IV (Fig. 2a). *Eriocaulon cinereum* belongs to sect. *Leucantherae* Fyson characterised by pale anthers and a smooth seed coat (Fyson 1919, 1921, 1922; Zhang 1999), and recognised by Ansari and Balakrishnan (1994, 2009) as their sect. XII. This group is represented by clade II of the ptDNA tree (Fig. 2a), and hence upheld by both morphological and molecular evidence.

Ma (1991, 1997) classified Chinese *Eriocaulon* into subgen. *Trimeranthus* of 27 species and subgen. *Eriocaulon* accommodating *E*. *decemflorum*. Although neither subgen. *Trimeranthus* nor most of its sections or series are supported in our results, it is noteworthy that *E. decemflorum* is retrieved as a single species lineage (Fig. 2a clade X). It was also placed as the only member of sect. *Nasmythia* by Zhang (1999) based on its dimerous flowers and seed coat structure.

Ansari and Balakrishnan (1994, 2009) proposed an infrageneric classification of the Indian species of *Eriocaulon* into twelve sections, primarily based on seed surface structure. These are mostly not supported by our molecular analysis. For instance, *E*. *nepalense*, *E*. *parviflorum* (Fyson) R. Ansari & N.P. Balakr. and *E*. *xeranthemum* Mart. are grouped in their sect. III, but are scattered in clades I, XI and XII of the ptDNA tree (Fig. 2a). Similarly, *E*. *truncatum* and *E*. *hamiltonianum* and are grouped in their sect. VII but are here placed in clades XI and XII, respectively (Fig. 2a).

Zhang (1999) carried out a morphology-based cladistic analysis of the 71 East Asian species studied. The resulting cladograms divided the species into four groups. None of these groups are supported in our ptDNA and PHYC trees (Fig. 2). However, noteworthy in our results is the sister relationship between clade I and the rest of *Eriocaulon* (Fig. 2). Clade I accommodates relatively robust and large species, i.e. *E*. *australe* R.Br., *E*. *cuspidatum* Dalzell and *E*. *sexangulare* L. Zhang (1999) placed *E. australe* and *E. sexangulare* in sect. *Heterochiton* Ruhland. Still, clades II–XII contain morphologically similar species, such as *E*. *rufum* Lecomte, *E*. *schimperi* Körn. ex Ruhland and *E*. *ubonense* Lecomte.

### Species distribution and taxonomy

Some species of *Eriocaulon* are known to have a wide distribution in the Old World tropics, such as *E*. *cinereum* and *E*. *setaceum* (Cook 1996, 2004). In the present study, *E*. *cinereum* is divided into two lineages, one from Africa and the other from Asia, although both fall within clade II (Fig. 2a). Similarly, samples of *E*. *setaceum* from Africa and Asia showed genetic variation (Fig. 2a clade XII). On the other hand, no significant differentiations are observed among samples of *E*. *truncatum* from Africa and Asia (Fig. 2a clade XI). Species such as *E. cinereum* and *E. truncatum* are common in rice fields (Cook 1996), probably contributing to their widespread distribution around the world.

Clade I includes a subclade of 12 accessions of *Eriocaulon australe* and *E. sexangulare*. Prajaksood et al. (2012) reduced *E. australe* to a variety of *E. sexangulare* (*E. sexangulare* var. *australe* (R.Br.) Praj. & J.Parn.). These taxa differ in *E. australe* having hairy leaves, sheaths, involucral bracts and receptable (Prajaksood et al. 2012; Zhang 1999). From our results it appears that this character may not be phylogenetically informative, and therefore, the varietal status of *E. australe* is supported.

Clade VII comprises *Eriocaulon alpestre* Hook.f. & Thomson ex Körn., *E*. *buergerianum* Körn., *E*. *sikokianum* Maxim., *E*. *hondoense*, *E*. *miquelianum* and *E*. *taquetii* with limited genetic variation among samples (Fig. 2 clade VII). This clade corresponds to subgen. *Spathopeplus* sect. *Apoda* of Zhang (1999), a mainly Asian group of species with female sepals connate into a spathe and seeds with T-shaped projections. Our results reflect the taxonomic complexity of this group in Japan, e.g. *E*. *sikokianum* Maxim. (accepted name: *E. miquelianum* Körn.) and *E*. *hondoense* (accepted name: *E. taquetii* Lecomte), while *E*. *buergerianum*_TCMK_493_K needs critical re-identification because this species is clearly diagnosed by floral morphology (Ma et al. 2000; Miyamoto 2015). Similarly, samples from Africa in clade XII show no clear-cut phylogenetic difference based on the markers used in this study: *E*. *burttii* S.M.Phillips, *E*. *crassiusculum* Lye, *E*. *deightonii* Meikle, *E*. *maronderanum* S.M. Phillips, *E*. *mutatum* N.E. Br, *E*. *nigericum* Meikle, *E*. *porembskii* S.M. Phillips & Mesterházy, *E*. *remotum* Lecomte and *E*. *transvaalicum* N.E. Br. However, these species are all distinguishable morphologically by floral structure and seed coat patterning (e.g. Phillips 1998, 2010, 2011). Further phylogenetic work is required to investigate the morphology-based hypotheses for species delimitation in Asian and African species of *Eriocaulon*.

Several of the 20 samples of *Eriocaulon* from Cambodia are phylogenetically unique and distinguished from other known species in both ptDNA and PHYC trees (Fig. 2). An in-depth morphological analysis may reveal whether the collections belong to undescribed species.

### Evolutionary history of *Eriocaulon*

Our results show that *Eriocaulon* originated in the late Paleogene to early Neogene (Fig. 3), and most species diversity originated in the last 10 mya. With *Eriocaulon* occurring in (permanent or ephemeral) wetlands across the tropics, the increased speciation during this time may be due to drift arising from fragmentation of suitable habitats associated with aridification since mid-Miocene. During the same period, Poales lineages like Cyperaceae and Poaceae that evolved adaptation to aridification (e.g. C4 photosynthesis, growing in non-wetland habitats) exhibited increased diversification (Bouchenak-Khelladi et al. 2014).

## Future perspectives

For the time being, we refrain from suggesting a new infrageneric classification of this polymorphic and widespread genus until the morphological characters used in previous studies (e.g. seed surface structure, anther colour, floral structure; Ansari and Balakrishnan 1994, 2009; Zhang 1999) can be thoroughly investigated for their phylogenetic informativeness. Further phylogenetic studies, particularly focusing on less well sampled regions such as the Neotropics, will help provide a more global overview of the relationships in *Eriocaulon* and may enable suggesting the first global infrageneric classification. Further research may also aid towards understanding closely related species groups in Africa and Asia. This study should be seen as a step towards achieving the aim of a natural classification.

## Electronic supplementary material

Below is the link to the electronic supplementary material.

Supplementary material 1 (PDF 592 kb)
