# Supplementary material for: First molecular phylogenetic insights into the evolution of Eriocaulon (Eriocaulaceae, Poales)
Source: J Plant Res. 2019 Aug 5;132(5):589–600. doi: 10.1007/s10265-019-01129-3 (PMC6713687; doi:10.1007/s10265-019-01129-3)
Supplement: Supplementary file 1 — Supplementary material 1 (PDF 592 kb) [file 10265_2019_1129_MOESM1_ESM.pdf]

Electronic supplementary materials

Title:

First molecular phylogenetic insights into the evolution of *Eriocaulon* (Eriocaulaceae, Poales)

Authors:

Isabel Larridon · Norio Tanaka · Yuxi Liang · Sylvia M. Phillips · Anders S. Barfod · Seong-Hyun Cho · Stephan W. Gale · Richard W. Jobson · Young-Dong Kim · Jie Li · A. Muthama Muasya · John A.N. Parnell · Amornrat Prajaksood · Kohtaroh Shutoh · Phetlasy Souladeth · Shuichiro Tagane · Nobuyuki Tanaka · Okihito Yano · Attila Mesterházy · Mark F. Newman · Yu Ito

Journal:

Journal of Plant Research

Corresponding author:

Isabel Larridon (B.A. Krukoff Curator of African Botany)
Royal Botanic Gardens, Kew, Richmond, Surrey, TW9 3AE, United Kingdom
Phone: +44 (0)20 8332 5271
Fax: n.a.
Email: i.larridon@kew.org

Content:

Tables S1–S2

Table S1. Overview of infrageneric classifications of *Eriocaulon*. Currently accepted names (WCSP 2019) are provided in parentheses. Taxa sampled in the molecular phylogenetic study are indicated with an asterisk (*) next to the name used in the phylogenetic tree.

Mueller (1859)	Fyson (1919, 1921, 1922)	Ma (1991, 1997)	Ansari and Balakrishnan (1994, 2009)	Zhang (1999)
**Sect. *Eriocaulon***	**Sect. *Setaceae* Fyson**	**Subgen. *Trimeranthus* Nakai**	**Sect. I**	**Subgen. *Trimeranthus* Nakai**
E. ciliiflorum F.Muell. (*E. cinereum* R.Br.*)	*E. intermedium* Körn. (*E. setaceum* L.*)	**Sect. *Macrocaulon* Ruhland**	*E. alpestre* Hook.f. & Thomson ex Körn.*	**Sect. *Macrocaulon* Ruhland**
E. concretum F.Muell.	*E. setaceum* L.*	**Ser. *Tmetopsis* Ruhland**	**Sect. II**	*E. setaceum* L.*
E. lividum F.Muell.	**Sect. *Simplices* Fyson**	*E. acutibracteatum* W.L.Ma	*E. helferi* Hook.f.	**Sect. *Simplices* Fyson**
E. monoscapum F.Muell.	*E. achiton* Körn.	*E. angustulum* W.L.Ma (*E. nantoense* Hayata*)	*E. rhodae* Fyson (*E. robustobrownianum* Ruhland)	*E. achiton* Körn.
E. spectabile F.Muell.	*E. barba-caprae* Fyson	*E. australe* R.Br.*	**Sect. III**	*E. boni* Lecomte
E. tortuosum F.Muell.	*E. collettii* Hook.f.	*E. brownianum* Mart.	*E. ansarii* Pradeep & Sunil	*E. brownianum* Mart.
**Sect. *Dimorphogyne* F.Muell.**	*E. dianae* Fyson (*E. heterolepis* Steud.)	*E. henryanum* Ruhland*	*E. balakrishnanii* Punekar, Lakshmin. & Vasudeva Rao	*E. collinum* Hook.f. (*E. odoratum* Dalzell*)
E. heterogynum F.Muell.	*E. duthiei* Hook.f.	*E. nantoense* Hayata*	*E. brownianum* Mart.	*E. ermeiense* W.L.Ma ex Z.X.Zhang
	E. geoffreyi Fyson (*E. pectinatum* Ruhland)	*E. pullum* T.Koyama (*E. nepalense* J.D.Prescott ex Bong* var. *nepalense*)	*E. devendranii* Vijaya Sankar, K.Ravik. & Ganesh Babu	*E. eglandulatum* Z.X.Zhang (*E. infirmum* Steud.* var. *infirmum*)
	E. gregatum Körn.	*E. rockianum* Hand.-Mazz.	*E. ensiforme* C.E.C.Fisch.	*E. heterolepis* Steud.
	E. luzulifolium Mart.* (*E. nepalense* var. *luzulifolium* (Mart.) Praj. & J.Parn.*)	***E. schochianum* Hand.-Mazz.**	*E. heterolepis* Steud.	*E. kunmingense* Z.X.Zhang
	E. nepalense J.D.Prescott ex Bong.*	*E. senile* Honda (*E. nepalense* J.D.Prescott ex Bong* var. *nepalense*)	*E. hookerianum* Stapf	*E. laosense* Moldenke*
	E. pumilio Hook.f. (*E. nepalense* var. *luzulifolium* (Mart.) Praj. & J.Parn.*)	*E. setaceum* L.*	*E. karnatakense* S.P.Gaikwad, Sardesai, U.S.Yadav & S.R.Yadav	*E. leianthum* W.L.Ma
	E. quinquangulare L.	*E. sexangulare* L.*	*E. kolhapurense* S.P.Gaikwad, Sardesai & S.R.Yadav	*E. luzulifolium* Mart.* (*E. nepalense* var. *luzulifolium* (Mart.) Praj. & J.Parn.*)

E. roseum Fyson	*E. sollyanum* Royle	*E. lanceolatum* Miq. ex Steud.	*E. nantoense* Hayata*
E. sedgwickii Fyson	*E. yaoshanense* Ruhland (*E. fluviatile* Trimen*)	*E. nepalense* J.D.Prescott ex Bong.*	*E. nepalense* J.D.Prescott ex Bong*
E. thwaitesii Körn.	**Ser. *Leiantha* W.L.Ma**	*E. odoratum* Dalzell*	*E. oryzetorum* Mart.
E. trilobum Buch.-Ham. Ex Körn. (*E. sollyanum* Royle)	*E. bilobatum* W.L.Ma (*E. kunmingense* Z.X.Zhang)	*E. oryzetorum* Mart.	*E. parvum* Körn.*
E. trimenii Hook.f.	*E. leianthum* W.L.Ma	*E. parviflorum* (Fyson) R.Ansari & N.P.Balakr.*	*E. pseudonepalense* Satake (*E. nepalense* var. *luzulifolium* (Mart.) Praj. & J.Parn.*)
E. truncatum Buch.-Ham. ex Mart.*	*E. zollingerianum* Körn.*	*E. richardianum* (Fyson) R.Ansari & N.P.Balakr.	*E. quinquangulare* L.
E. xeranthemum Mart.*	**Ser. *Disepala* Satake**	*E. robustobrownianum* Ruhland	*E. robustobrownianum* Ruhland
**Sect. *Hirsutae* Fyson**	*E. merrillii* Ruhland ex Perkins (*E. truncatum* Buch.-Ham. Ex Mart.*)	*E. santapaui* Moldenke	*E. rockianum* Hand.-Mazz.
E. brownianum Mart.	*E. sclerophyllum* W.L.Ma	*E. sharmae* R.Ansari & N.P.Balakr.	*E. schochianum* Hand.-Mazz.
E. gracile Mart.	**Sect. *Leucocephala* Nakai**	*E. sivarajanii* R.Ansari & N.P.Balakr.	*E. smitinandii* Moldenke
E. lanceolatum Miq. Ex Steud.	*E. cinereum* R.Br.*	*E. stellulatum* Körn.	*E. sollyanum* Royle
E. rhodae Fyson	*E. echinulatum* Mart.*	*E. trilobum* Buch.-Ham. Ex Körn. (*E. sollyanum* Royle)	*E. thailandicum* Moldenke*
E. robusto-brownianum Ruhland	*E. taishanense* F.Z.Li	*E. thwaitesii* Körn.	*E. wightianum* Mart.*
E. wightianum Mart.*	**Sect. *Spathopeplus* Nakai**	*E. vasudevanii* R.Ansari & N.P.Balakr.	*E. xenopodion* T.Koyama
**Sect. *Anisopetalae* Fyson**	**Ser. *Miqueliana* Satake**	*E. xeranthemum* Mart.*	*E. xeranthemum* Mart.*
E. atratum Körn.	*E. buergerianum* Körn.*	**Sect. IV**	**Sect. *Anisopetalae* Fyson**
E. ceylanicum Körn.	*E. sikokianum* Maxim.* (*E. miquelianum* Körn.*)	*E. capillus-naiadis* Hook.f. (*E. setaceum* L.)	*E. bassacense* Moldenke
E. cristatum Mart.*	**Ser. *Robustiora* W.L.Ma**	***E. setaceum* L.**	*E. cristatum* Mart.*
E. longicuspis Hook.f.	*E. alpestre* Hook.f. & Thomson ex Körn.*	**Sect. V**	*E. eberhardtii* Lecomte

E. odoratum Dalzell*

E. robustum Steud.

Sect. *Scariosae* Fyson

E. edwardii Fyson

E. hamiltonianum Mart.*

E. oryzetorum Mart.

Sect. *Cristato-sepalae* Fyson

E. cuspidatum Dalz.*

E. echinulatum Mart.*

E. elenorae Fyson

E. margaretae Fyson

E. minutum Hook.f.

E. sexangulare L.*

E. stellulatum Körn.

E. thomasi Fyson

Sect. *Connato-sepalae* Fyson

E. alpestre Hook.f. & Thomson ex Körn.*

Sect. *Leucantherae* Fyson

E. breviscapum Körn.*

E. chinorossicum Kom.

E. glabripetalum W.L.Ma

E. minusculum Moldenke

E. robustium (Maxim.) Makino (*E. alpestre* Hook.f. & Thomson ex Körn.)

Ser. *Manshanensia* W.L.Ma

E. faberi Ruhland

E. mangshanense W.L.Ma

Subgen. *Eriocaulon*

E. decemflorum Maxim.*

E. longifolium Nees ex Kunth (*E. willdenovianum* Moldenke)

E. peninsulare Punekar & Lakshmin.

E. sexangulare L.*

Sect. VI

E. achiton Körn.

E. anshiense Punekar, Malpure & Lakshmin.

E. cuspidatum Dalzell*

E. duthiei Hook.f.

E. eurypeplon Körn.

E. fysonii R.Ansari & N.P.Balakr.

E. koynense Punekar, Mungikar & Lakshmin.

E. sedgwickii Fyson

Sect. VII

E. cherrapunjanum R.Ansari & N.P.Balakr.

E. conicum (Fyson) C.E.C.Fisch.

E. cristatum Mart.*

E. edwardii Fyson

E. hamiltonianum Mart.*

E. longicuspe Hook.f.

E. henryanum Ruhland*

E. hookerianum Stapf

*E. kradungense** Satake (*E. ubonense* Lecomte* subsp. *ubonense*)

E. nautiliforme Lecomte*

E. siamense Lecomte*

E. tanakae Ruhland

Sect. *Heterochiton* Ruhland

E. australe R.Br.*

E. sexangulare L.*

E. willdenovianum Moldenke

Sect. *Disepala* (Satake) Z.X.Zhang

E. dipsacoides Satake (*E. escape* B.F.Hansen)

E. echinulatum Mart.*

E. hamiltonianum Mart.*

E. nigrum Lecomte

E. pseudotruncatum Z.X.Zhang

E. thwaitesii Körn.

E. truncatum Buch.-Ham. Ex Mart.*

E. zollingerianoides

E. fluviatile Trimen*

E. horsley-kondae Fyson

E. miserum Körn.

E. rivulare Dalzell

E. sieboldianum Siebold & Zucc. ex Steud. (*E. cinereum* R.Br.*)

E. martianum Wall. ex Körn. (*E. quinquangulare* L. subsp. *quinquangulare*)

E. palghatense R.Ansari & N.P.Balakr.

E. quinquangulare L.

E. raipurense K.K.Khanna & Mudgal & An.Kumar

E. ramnadense R.Ansari & N.P.Balakr.

E. truncatum Buch.-Ham. ex Mart.*

Sect. VIII

E. collettii Hook.f.

E. nairii Chandrab. & V.Chandras.

E. robustum Steud.

Sect. IX

E. elenorae Fyson

E. margaretae Fyson

E. meeboldii R.Ansari & N.P.Balakr.

E. minutum Hook.f.

Sect. X

E. apetalum Punekar, Malpure & Lakshmin.

E. kanarense Punekar, Watve & Lakshmin.

Z.X.Zhang (*E. hamiltonianum* Mart. var. *hamiltonianum*)

E. zollingerianum Körn.*

Sect. Leucantherae Fyson

E. barbeyanum Ruhland (*E. fluviatile* Trimen*)

E. cinereum R.Br.*

E. taishanense F.Z.Li

E. tonkinense Ruhland* (*E. fluviatile* Trimen*)

Sect. Nasmythia Huds.

E. decemflorum Maxim.*

Subgen. *Spathopeplus* Körn.

Sect. Apoda (Satake) Z.X.Zhang

***E. alpestre* Hook.f. & Thomson ex Körn.**

E. atriodes Satake (*E. miquelianum* Körn.*)

E. atrum Nakai

E. buergerianum Körn.*

E. faberi Ruhland

E. hondoense Satake* (*E. taquetii* Lecomte*)

E. japonicum Körn.

E. kusiroense Miyabe & Kudô ex Satake (*E. sachalinense* var. *kusiroense*

E. maharashtrense Punekar & Lakshmin.

E. pectinatum Ruhland

E. rajendrababui R.Ansari & N.P.Balakr.

E. sahyadricum Punekar, Malpure & Lakshmin. (*E. stellulatum* Körn.)

Sect. XI

E. tuberiferum A.R.Kulk. & Desai

Sect. XII

E. barbeyanum Ruhland (*E. fluviatile* Trimen*)

E. breviscapum Körn.*

E. cinereum R.Br.*

E. cookei Punekar, Malpure & Lakshmin.

E. dalzellii Körn.

E. fluviatile Trimen*

E. leucomelas Steud.

E. miserum Körn.

E. mitophyllum Hook.f. (*E. fluviatile* Trimen*)

E. panagudianum R.Ansari & N.P.Balakr.

E. ratnagiricus S.R.Yadav, S.P.Gaikwad & Sardesai

(Miyabe & Kudô ex Satake) T.Koyama ex Miyam.)

E. liberisepalum Z.X.Zhang

E. minusculum Moldenke

***E. miquelianum* Körn.**

E. monococcon Nakai

E. nanellum Ohwi (*E. takae* Koidz.)

E. sachalinense Miyabe & Nakai

E. sekimotoi Honda (*E. taquetii* Lecomte*)

E. sphagnicola Ohwi (*E. sachalinense* var. *kusiroense* (Miyabe & Kudô ex Satake) T.Koyama ex Miyam.)

E. takae Koidz.

E. tenuissimum Nakai (*E. miquelianum* Körn.)

E. zyotanii Satake

Sect. Macropoda (Satake) Z.X.Zhang

E. heleocharioides Satake*

Sect. Nudicuspa Z.X.Zhang

E. nudicuspe Maxim.

	E. redactum Ruhland
	E. ritchieanum Ruhland
	E. talbotii R.Ansari & N.P.Balakr.

Table S2. Specimen and voucher information for *Eriocaulon* and its outgroup taxa included in this study. Sequences generated in the present study are underlined. Herbarium acronyms are in accordance with Index Herbariorum (http://sciweb.nybg.org/science2/IndexHerbariorum.asp).

Taxon	Voucher information	Locality	Lab ID	*matK*	*rbcL*	*rpoB*	*rpoC1*	*PHYC*
OUTGROUP								
Xyris Gronov. ex L. sp.	Chase, M.W. s.n. (NCU)	n/a		-	-	-	-	KP083152
Syngonanthus angolensis H.E.Hess	Goyder, D.J. 8359 (K)	Angola	TD5169	LC461615	LC461623	LC461636	LC461644	LC461650
Syngonanthus chrysanthus (Bong.) Ruhland (ID unsure GenBank)	Ames, M. 10/15/2009	n/a		-	-	HQ182897	-	-
Syngonanthus flavidulus (Michx.) Ruhland	Abbott 22378 (FLAS)	n/a		KJ773197	-	-	-	-
Syngonanthus peruvianus Ruhland	Jorgensen, P. M., Luteyn, J. L. & Romoleroux, K. 92740 (AAU)	Ecuador	TD5212	LC461616	LC461624	LC461637	LC461645	-
Paepalanthus muscosus Körn.	Laegaard, S. 54916 (AAU)	Ecuador	TD5207	LC461627	-	LC461639	-	-
Paepalanthus obnatus Tissot-Sq.	Madsen, J. E. 7154 (AAU)	Ecuador	TD5218	LC461618	LC461626	LC461638	-	-
Paepalanthus pilosus (Kunth) Kunth	Laegaard, S. 53217 (AAU)	Ecuador	TD5198	LC461619	LC461628	-	-	-
Tonina fluviatilis Aubl. (ID unsure GenBank)	n/a	n/a		-	AY123237	-	-	-
Mesanthemum albidum Lecomte	Molmou, D. 919 (K)	Guinea-Conakry	TD5094	-	LC461630	-	-	-
Mesanthemum glabrum Kimp.	Goyder, D.J. 8358 (K)	Angola	TD5174	-	LC461635	LC461643	LC461649	LC461653
Mesanthemum prescottianum (Bong.) Körn.	Couch, C.A. 754 (K)	Guinea-Conakry	TD5095	LC461620	LC461632	LC461641	LC461647	LC461651
Mesanthemum radicans (Benth.) Körn.	van der Burgt, X.M. 1732 (K:001061936)	Sierra Leone	TD5059	LC461621	LC461633	LC461642	LC461648	LC461652
INGROUP								
E. afzelianum Wikstr. ex Körn.	Fofana, F. 233 (K:000749971)	Guinea-Conakry	TD5064	-	LC485810	-	-	-
E. asteroids S.M.Phillips	Pollard, B.J. 700 (K:000746173)	Cameroon	TD5105	-	LC485811	-	-	-
E. australe R.Br.	Cho et al. CB-0568 (HHU)	Cambodia	CB0568	LC485678	LC485812	LC486730	LC484925	-

E. australe R.Br.	Cho et al. CB-0767 (HHU)	Cambodia	CB0767	LC485679	LC485813	LC486731	LC484926	-
E. australe R.Br.	Ito, Y. YI2344 (TNS)	Australia	YI2344	LC485761	LC485926	LC485617	LC484949	LC484480
E. sexangulare L.	Tanaka, N. s.n. (TNS)	Singapore	TD5253	LC485680	LC485814	LC486732	LC484927	LC484447
E. sexangulare L.	Ito, Y. YI2299 (TNS)	Malaysia	YI2299	LC485681	LC485815	LC486733	LC484928	LC484448
E. wightianum Mart.	Toyama, H. et al. 2516 (TNS)	Cambodia	TD4995	LC485682	LC485816	LC486734	LC484929	-
E. bongense Engl. & Ruhland	MABEN202 (BP)	Benin	TD5437	LC485683	LC485817	-	-	-
E. breviscapum Körn.	Tagane, S. et al. 4317 (TNS)	Cambodia	TD4998	-	-	LC486735	LC484930	-
E. sp. 08	Won et al. HW-9169 (HHU)	Cambodia	HW9169	LC485684	LC485818	-	-	LC484449
E. sp. Cambodia	Toyama, H. et al. 1759 (TNS)	Cambodia	TD4994	-	LC485819	LC486736	-	-
E. carsonii F.Muell.	Ito, Y. YI2309 (TNS)	Australia	YI2309	LC485685	LC485820	-	-	-
E. carsonii F.Muell.	Ito, Y. YI2312 (TNS)	Australia	YI2312	LC485686	LC485821	LC485330	LC484931	LC484450
E. carsonii F.Muell.	Ito, Y. YI2324 (TNS)	Australia	YI2324	LC485687	LC485822	LC485331	-	LC484451
E. cinereum R.Br.	Porembski, S. 2999 (K)	Ivory Coast	TD5068	LC485688	LC485823	-	-	-
E. cinereum R.Br.	van der Burgt, X.M. 1357 (K:000023442)	Sierra Leone	TD5069	LC485689	LC485824	LC485332		-
E. cinereum R.Br.	Mesterhàzy, A. MAGUI215 (BP)	Guinea Conakry	TD5442	-	LC485825	-	-	LC484452
E. sp. Burkina Faso	Laegaard, S., Mipro Hien and H. Sangaré 21226 (AAU)	Burkina Faso	TD5187	-	LC485826	-	-	-
E. cinereum R.Br.	Enju, C. CED076 (TNS)	Japan: Aichi	CED076	LC485690	LC485827	-	-	LC484453
E. cinereum R.Br.	H. Fukuhara FOK-078924 (TNS:01141509)	Japan	TD5036	LC485691	LC485828	LC485333	-	-
E. cinereum R.Br.	Kato, S. YI1120 (TNS)	Japan: Nagasaki	YI1120	-	LC485829	-	-	-
E. sp. Australia (cf. *E. cinereum* R.Br.)	Ito, Y. YI2311 (TNS)	Australia	YI2311	LC485692	LC485830	LC485334	-	LC484454
E. sp. Japan (cf. *E. cinereum* R.Br.)	Enju, C. CED092 (TNS)	Japan: Kanagawa	CED092	LC485693	LC485831	-	-	-
E. sp. Myanmar (cf. *E.*	Tanaka, N. MY1884 (TNS)	Myanmar	NT102	LC485694	LC485832	LC485335	LC484932	LC484455

cinereum R.Br.)									
E. sp. Myanmar (cf. *E. cinereum* R.Br.)	Tanaka, N. MY1875 (TNS)	Myanmar	NT93	LC485695	LC485833	LC485336	LC484933	LC484456
E. parviflorum (Fyson) R.Ansari & N.P.Balakr.	Ito, Y. YI1194 (TNS)	India	YI1194	-	-	LC485559	LC484934	-
E. cuspidatum Dalzell	Ito, Y. YI1202 (TNS)	India	YI1202	-	-	LC485560	-	-
E. sp. 06	Cho et al. CB-0768 (HHU)	Cambodia	CB0768	LC485696	LC485834	LC485561	-	-
E. compressum Lam.	Abbott 18806 (FLAS)	U.S.A.		KJ772760	KJ773484	-	-	-
E. decangulare L.	Abbott 22496 (FLAS)	U.S.A.		KJ772761	KJ773485	-	-	-
E. decemflorum Maxim.	Mitsuda et al. 94178 (TNS:722501)	Japan	TD5013	-	LC485835	-	-	-
E. dregei Hochst.	Gotze, AR 121 (PRE)	South Africa	TD5300	LC485697	LC485836	LC485562	-	-
E. echinulatum Mart.	Cho et al. CB-0592 (HHU)	Cambodia	CB0592	LC485698	LC485837	LC485563	-	-
E. echinulatum Mart.	Souladeth, P. PS365 (FOF)	Laos	PS365	LC485699	LC485838	LC485564	-	-
E. echinulatum Mart.	Souladeth, P. PS447 (FOF)	Laos	PS447	LC485700	LC485839	LC485565	LC484935	LC484457
E. fluviatile Trimen	Gale, S.W. et al. HNL-KFBG 934 (HNL)	Laos	HNL0934	LC485701	LC485840	LC485566	LC484936	LC484458
E. fluviatile Trimen	Souladeth, P. PS378 (FOF)	Laos	PS378	LC485702	LC485841	LC485567	-	-
E. sp. 10	Won et al. HW-8707 (HHU)	Cambodia	HW8707	LC485703	LC485842	-	-	LC484459
E. sp. 10	Won et al. HW-8816 (HHU)	Cambodia	HW8816	LC485704	LC485843	-	-	LC484460
E. sp. Myanmar	Murata et al. 040646 (TI)	Myanmar	M040646	LC485705	LC485844	LC485568	-	-
E. hamiltonianum Mart.	Prajaksood, A. AP405 (KKU)	Thailand	AP405	LC485706	LC485845	LC485569	-	-
E. hamiltonianum Mart.	Gale, S.W. et al. HNL-KFBG 876 (HNL)	Laos	HNL0876	LC485707	LC485846	LC485570	-	LC484461
E. sp. Laos (cf. *E. hamiltonianum* Mart.)	Fischer, G. et al. SG1555 (HNL/TNS)	Laos	SG1555	-	LC485847	LC485571	-	-
E. sp. Myanmar (cf. *E. hamiltonianum* Mart.)	Tanaka, N. MY1883 (TNS)	Myanmar	NT101	LC485708	LC485848	LC485572	-	-
E. heleocharioides Satake	Tanaka, N. TD3741 (TNS)	Japan	TD3741	LC485709	-	-	-	-
E. heleocharioides Satake	Tanaka, N. TD3769 (TNS)	Japan	TD3769	LC485710	-	LC485573	-	-

E. heleocharioides Satake	Tanaka, N. TD3961 (TNS)	Japan	TD3961	LC485711	LC485849	-	-	LC484462
E. alpestre Hook.f. & Thomson ex Körn. (ID unsure)	KUN:0808382	China: Yunnan	YI2242	-	-	LC485574	LC484937	-
E. henryanum Ruhland	KUN:0987134	China: Yunnan	YI2234	-	-	LC485575	LC484938	-
E. humboldtii Kunth	n/a	South America		-	AY123236	-	-	-
E. infirmum Steud.	Souladeth, P. PS372 (FOF)	Laos	PS372	LC485712	LC485850	LC485576	-	-
E. phuphanense Praj. & J.Parn.	Souladeth, P. PS463 (FOF)	Laos	PS463	LC485713	LC485851	LC485577	-	-
E. inyangense Arw.	Goyder, D.J. et al. 3910 (K:000334401)	Tanzania	TD5140	-	LC485852	LC485578	-	-
E. lanatum H.E.Hess	Goyder, D.J. 8202 (K)	Angola	TD5170	-	LC485853	-	-	-
E. lanatum H.E.Hess	Goyder, D.J. 8369 (K)	Angola	TD5171	-	LC485854	LC485579	-	-
E. laniceps S.M.Phillips	Bidgood, S. et al. 5496 (K:000190726)	Tanzania	TD5141	-	LC485855	LC485580	-	-
E. laniceps S.M.Phillips	Bidgood, S. et al. 4528 (K)	Tanzania	TD5142	-	LC485856	-	-	-
E. lanigerum Lecomte	Souladeth, P. PS450 (FOF)	Laos	PS450	LC485714	LC485857	LC485581	-	-
E. lanigerum Lecomte	Gale, S.W. et al. HNL-KFBG 877 (HNL)	Laos	HNL0877	LC485715	LC485858	LC485582	-	LC484463
E. sp. 04	Cho et al. CB-0581 (HHU)	Cambodia	CB0581	-	LC485859	LC485583	-	-
E. laosense Moldenke	Cho et al. CB-0663 (HHU)	Cambodia	CB0663	LC485716	LC485860	-	-	LC484464
E. latifolium Sm.	James, M.S. 113 (K:000024050)	Sierra Leone	TD5074	LC485717	LC485861	LC485584	-	-
E. latifolium Sm.	van der Burgt, X.M. 1252 (K:001061388)	Guinea-Conakry	TD5076	LC485718	LC485862	LC485585	-	LC484465
E. latifolium Sm.	Mesterhàzy, A. MALIB258 (BP)	Liberia	TD5449	LC485719	LC485863	LC485586	-	-
E. latifolium Sm.	Fofana, F. 2015 (K:000749970)	Guinea-Conakry	TD5075	-	LC485864	-	-	-
E. microcephalum Kunth	Laegaard, S. 51867 (AAU)	Ecuador	TD5189	LC485720	LC485865	LC485587	-	-
E. microcephalum Kunth	Balslev, H., Paz, G. & Renner, S. S. 69151 (AAU)	Ecuador	TD5209	-	LC485866	-	-	-
E. microcephalum Kunth	n/a	n/a		-	L10252	-	-	-
E. sp. Ecuador (cf. *E. microcephalum* Kunth)	Laegaard, S. 53137 (AAU)	Ecuador	TD5197	LC485721	LC485867	-	-	-

E. sp. Ecuador (cf. *E. microcephalum* Kunth)	Laegaard, S. 54153 (AAU)	Ecuador	TD5204	-	LC485868	-	-	-
E. sp. Ecuador (cf. *E. microcephalum* Kunth)	Laegaard, S. 54763 (AAU)	Ecuador	TD5205	-	LC485869	-	-	-
E. sp. Ecuador (cf. *E. microcephalum* Kunth)	Laegaard, S. 54883 (AAU)	Ecuador	TD5206	-	LC485870	-	-	-
E. sp. Ecuador (cf. *E. microcephalum* Kunth)	Laegaard, S. 19384 (AAU)	Ecuador	TD5216	LC485722	LC485871	-	-	-
E. sp. Ecuador (cf. *E. microcephalum* Kunth)	Jane, E. Madsen & C. Chmbo 8506 (AAU)	Ecuador	TD5234	-	LC485872	-	-	-
E. nantoense Hayata	Fischer, G. FS7555 (KFBG)	China: Hong Kong	FS7555	LC485723	LC485873	LC485588	-	LC484466
cf. *E. nantoense* Hayata	Gale, S.W. et al. HNL-KFBG 938 (HNL)	Laos	HNL0938	LC485724	LC485874	LC485589	-	LC484467
E. nautiliformoides Praj. & J.Parn.	Prajaksood, A. AP361 (KKU)	Thailand	AP361	-	LC485875	LC485590	-	-
E. nautiliformoides Praj. & J.Parn.	Prajaksood, A. AP366 (KKU)	Thailand	AP366	LC485725	LC485876	LC485591	-	LC484468
E. nautiliformoides Praj. & J.Parn.	Prajaksood, A. AP370 (KKU)	Thailand	AP370	LC485726	LC485877	LC485592	-	-
E. nautiliformoides Praj. & J.Parn.	Prajaksood, A. AP372 (KKU)	Thailand	AP372	LC485727	LC485878	LC485593	-	-
E. sp. Thai	Prajaksood, A. AP387 (KKU)	Thailand	AP387	LC485728	LC485879	-	-	-
E. nepalense J.D.Prescott ex Bong. (ID unsure)	Souladeth, P. PS353 (FOF)	Laos	PS353	LC485729	LC485880	LC485594	-	LC484469
E. truncatum (ID unsure)	Cho et al. CB-2757 (HHU)	Cambodia	CB2757	-	LC485881	LC485595	-	-
E. nepalense J.D.Prescott ex Bong.	Naito s.n. (TNS:748336)	Japan	TD5014	-	LC485921	-	-	-
E. nepalense J.D.Prescott ex Bong.	Koga, Y. et al. 14885 (TNS:729621)	Japan	TD5015	-	LC485882	-	-	-
E. setaceum L.	Prajaksood, A. AP419 (KKU)	Thailand	AP419	LC485730	LC485883	-	-	-
E. odoratum Dalzell	Prajaksood, A. AP440 (KKU)	Thailand	AP440	-	LC485884	-	-	-
E. odoratum Dalzell	Prajaksood, A. AP441 (KKU)	Thailand	AP441	LC485731	LC485885	LC485596	-	LC484470

E. odoratum Dalzell	Prajaksood, A. AP445 (KKU)	Thailand	AP445	LC485732	LC485886	-	-	-
E. odoratum Dalzell	Prajaksood, A. AP446 (KKU)	Thailand	AP446	LC485733	LC485887	LC485597	-	-
E. odoratum Dalzell	Prajaksood, A. AP452 (KKU)	Thailand	AP452	-	LC485888	-	-	-
E. parnellii Praj. & Chantar.	Prajaksood, A. AP415 (KKU)	Thailand	AP415	LC485734	LC485889	-	-	-
E. nepalense var. *luzulifolium* (Mart.) Praj. & J.Parn.	Prajaksood, A. AP451 (KKU)	Thailand	AP451	-	LC485890	-	-	-
E. parvum Körn.	Tanaka, N. TD1324 (TNS)	Japan	TD1324	LC485735	LC485891	-	-	-
E. nepalense J.D.Prescott ex Bong.	Murata et al. 040640 (TI)	Myanmar	M040640	-	LC485892	-	-	-
E. petraeum S.M.Phillips & Burgt	Lopez Poveda, L. LLP299 (K)	Sierra Leone	LLP299	LC485736	LC485893	LC485598	LC484939	LC484471
E. petraeum S.M.Phillips & Burgt	Lopez Poveda, L. LLP300 (K)	Sierra Leone	LLP300	LC485737	LC485894	LC485599	-	LC484472
E. sulanum S.M.Phillips & Burgt	Kanu, K.M.T. 109 (K:000023837)	Sierra Leone	TD5101	LC485738	LC485895	-	-	-
E. nautiliforme Lecomte	Prajaksood, A. AP403 (KKU)	Thailand	AP403	LC485739	LC485896	-	-	-
E. phuphanoides Praj. & J.Parn.	Gale, S.W. et al. HNL-KFBG 936 (HNL)	Laos	HNL0936	LC485740	LC485897	LC485600	LC484940	LC484473
E. siamense var. *hexagynum* Praj. & Chantar.	Prajaksood, A. AP436 (KKU)	Thailand	AP436	LC485741	LC485898	LC485601	-	-
E. plumale N.E.Br.	Mesterhàzy, A. MAIVO251 (BP)	Ivory Coast	TD5460	LC485742	LC485899	LC485602	LC484941	-
E. plumale N.E.Br.	Pearce, L.J. 39 (K:000580619)	Guinea-Conakry	TD5083	LC485743	LC485900	LC485603	LC484942	LC484474
cf. E. deightonii Meikle (= *E.* sp. Sierra Leone)	Mesterhàzy, A. MASIE256 (BP)	Sierra Leone	TD5455	-	LC485901	-	-	-
E. nadjae S.M.Phillips	Mesterhàzy, A. MALIB199 (BP)	Liberia	TD5438	LC485744	LC485902	-	-	-
E. plumale N.E.Br.	Mesterhàzy, A. MAGUI214 (BP)	Guinea Conakry	TD5452	LC485745	LC485903	LC485604	LC484943	-
E. plumale N.E.Br.	Mesterhàzy, A. MAGUI212 (BP)	Guinea Conakry	TD5453	-	LC485904	-	-	-

E. plumale subsp. *jaegeri* (Moldenke) Meikle	Dörrstock, S. 186 (K)	Ivory Coast	TD5080	LC485746	LC485905	LC485605	LC484944	-
E. plumale subsp. *kindiae* (Lecomte) Meikle	Cheek, M. 16074 (K:000749438)	Guinea-Conakry	TD5082	LC485747	LC485906	-	LC484945	-
E. plumale N.E.Br.	Mesterhàzy, A. MAGUI194 (BP)	Guinea Conakry	TD5451	LC485748	LC485907	LC485606	LC484946	-
E. cryptocephalum S.M.Phillips & Mesterházy	Couch, C.A. 779 (K)	Guinea-Conakry	TD5098	LC485749	LC485908	-	-	-
E. pulchellum Körn.	Merklinger, F.F. 167 (K:000749332)	Guinea-Conakry	TD5084	LC485750	LC485909	LC485607	-	LC484475
E. pulchellum Körn.	Haba, P.M. 199 (K:000580618)	Guinea-Conakry	TD5085	LC485751	LC485910	LC485608	-	-
E. remotum Lecomte	Molmou, D. 561 (K:000749385)	Guinea-Conakry	TD5087	-	LC485911	-	-	-
E. rufum Lecomte	Mesterhàzy, A. MAGUI213 (BP)	Guinea Conakry	TD5448	-	LC485912	-	-	-
E. rufum Lecomte	Cheek, M. 18269 (K)	Guinea-Conakry	TD5088	-	LC485913	LC485658	-	-
E. scariosum Sm.	Ito, Y. YI2310 (TNS)	Australia	YI2310	LC485752	LC485914	LC485609	-	LC484476
E. scariosum Sm.	Ito, Y. YI2341 (TNS)	Australia	YI2341	LC485753	LC485915	LC485610	-	LC484477
E. scariosum Sm.	Ito, Y. YI2343 (TNS)	Australia	YI2343	-	LC485916	-	-	-
E. scariosum Sm.	Ito, Y. YI2345 (TNS)	Australia	YI2345	LC485754	LC485917	-	-	-
E. schimperi	Faden et al. 96-440 (K:3065)	Tanzania	K3065	LC485755	KC123361	LC485611	LC484947	KP083147
E. buergerianum Körn.	KUN:0774981	China: Yunnan	YI2237	LC485756	LC485918	-	-	-
E. cinereum R.Br.	Ito, Y. YI2346 (TNS)	Japan: Wakayama	YI2346	LC485757	LC485919	LC485612	LC484948	LC484478
E. cristatum Mart.	KUN:0771166	China: Yunnan	YI2236	LC485758	LC485920	LC485613	-	-
E. schochianum Hand.-Mazz.	Tagane, S. V5362 (DLU/TNS)	Viet Nam	TD5050	LC485759	LC485922	LC485614	-	LC484479
E. septangulare With. (= *E. aquaticum* (Hill) Druce)	n/a	n/a		AY952430	-	-	-	-
E. sessile Meikle	Mesterhàzy, A. MAGUI204 (BP)	Guinea Conakry	TD5454	-	LC485923	-	-	-

E. setaceum L.	Faden et al. 96-515 (K:3066)	n/a		KP083049	-	-	-	-
E. setaceum L.	Gale, S.W. et al. HNL-KFBG 933 (HNL)	Laos	HNL0933	LC485760	LC485924	LC485615	-	-
E. setaceum L.	Cheek, M. 18198 (K)	Guinea-Conakry	TD5089	-	LC485925	LC485616	-	-
E. sexangulare L.	Gale S.W. SG1372 (KFBG)	China: Hong Kong	SG1372	LC485762	LC485927	LC485618	LC484950	LC484481
E. sexangulare L.	Gale, S.W. SG1375 (KFBG)	China: Hong Kong	SG1375	LC485763	LC485928	-	-	LC484482
E. sexangulare L.	Ito, Y. YI2271 (TNS)	Japan: Okinawa	YI2271	LC485764	LC485929	LC485619	LC484951	LC484483
E. sexangulare L.	Ito, Y. YI2283 (TNS)	Japan: Okinawa	YI2283	LC485765	LC485930	LC485620	LC484952	LC484484
E. sp. Australia (cf. *E. sexangulare* L.)	YSL4415 (TNS)	Australia	TD5433	LC485766	LC485931	LC485621	LC484953	LC484485
E. sp. Macau (cf. *E. sexangulare* L.)	Liang, Y.-S. s.n. CED325 (TNS)	Macau	CED325	LC485767	LC485932	LC485622	LC484954	LC484486
E. siamense Moldenke	Prajaksood, A. AP420 (KKU)	Thailand	AP420	LC485768	LC485933	LC485623	-	-
E. siamense Moldenke	Prajaksood, A. AP429 (KKU)	Thailand	AP429	-	LC485934	-	-	-
E. siamense Moldenke	Prajaksood, A. AP425 (KKU)	Thailand	AP425	LC485769	LC485935	-	-	-
E. abyssinicum Hochst.	Faden, R.B. et al. 96/125 (K)	Tanzania	TD5122	LC485770	LC485936	LC485624	-	-
E. abyssinicum Hochst.	Smook, L 5070 (PRE)	South Africa	TD5296	LC485771	LC485937	LC485625	-	-
E. cinereum L.	Giess, W 10245 (PRE)	Namibia	TD5298	LC485772	LC485938	LC485626	-	-
E. mutatum N.E.Br.	Vlok, OH 1149 (PRE)	Namibia	TD5306	-	LC485939	-	-	-
E. welwitschii Rendle	Faden, R.B. et al. 96/497 (K)	Tanzania	TD5130	LC485773	LC485940	LC485627	-	-
E. welwitschii Rendle	Bidgood, S. et al. 5628 (K:000468796)	Tanzania	TD5131	LC485774	LC485941	-	-	-
E. burttii S.M.Phillips	Bidgood, S. et al. 7209 (K)	Tanzania	TD5134	-	LC486037	LC485628	-	LC484487
E. crassiusculum Lye	Drummond, R.B. & Hemsley, J.H. 4693 (K)	Uganda	TD5136	-	LC486038	-	-	-

E. deightonii Meikle	Cheek, M. 18258 (K)	Guinea-Conakry	TD5093	LC485775	LC486039	LC485629	-	-
E. maronderanum S.M.Phillips	Smith, P.P. 1712 (K)	Zambia	TD5160	-	LC486040	LC485630	-	-
E. mutatum var. *angustisepalum* (H.E.Hess) S.M.Phillips	Faden, R.B. et al. 96/127 (K)	Tanzania	TD5125	-	LC486041	LC485631	-	-
E. nigericum Meikle	Cheek, M. 16751 (K:000749331)	Guinea-Conakry	TD5079	LC485776	LC486042	-	-	-
E. porembskii S.M.Phillips & Mesterházy	Mesterhàzy, A. MAIVO238 (BP)	Ivory Coast	TD5458	LC485777	LC486043	LC485632	-	-
E. transvaalicum subsp. *dembianense* (Chiov.) S.M.Phillips	Friis, I. et al. 11944 (K)	Ethiopia	TD5119	LC485778	LC486044	LC485633	-	-
E. transvaalicum subsp. *hanningtonii* (N.E.Br.) S.M.Phillips	Cheek, M. 16798 (K:000749430)	Guinea-Conakry	TD5092	-	LC486045	-	-	-
E. sp. 01	Cho et al. CB-2736 (HHU)	Cambodia	CB2736	LC485779	LC486046	-	-	-
E. sp. Cambodia	Toyama, H. 2518 (TNS)	Cambodia	TD4997	LC485780	LC486047	-	-	-
E. sp. Cambodia	Fuse, K. 6353 (TNS)	Cambodia	TD5003	LC485781	LC486048	-	-	LC484488
E. sp. India	Ito, Y. YI1176 (TNS)	India	YI1176	-	LC486049	LC485634	-	-
E. cinereum L.	Ito, Y. YI1161 (TNS)	India	YI1161	-	LC486050	-	-	-
E. sp.	Pell et al. 647 (NY)	n/a		KF724337	-	-	-	-
E. sp. Laos	Gale, S.W. et al. HNL-KFBG 875 (HNL)	Laos	HNL0875	LC485782	LC486051	LC485635	-	LC484489
E. fluviatile Trimen	Gale, S.W. et al. HNL-KFBG 929 (HNL)	Laos	HNL0929	LC485783	LC486052	LC485636	LC484955	-
E. sp. Laos	S. Koi et al. LKF-126 (TNS:01140737)	Laos	TD5044	LC485784	LC486053	-	-	-
E. sp. Myanmar	Tanaka et al. 021704 (TI)	Myanmar	M021704	-	LC486054	LC485637	-	-
E. sp. Myanmar	Murata et al. 041205 (TI)	Myanmar	M041205	-	-	LC485638	LC484956	-
E. sp. Thai (cf. *E. ubonense* f. *kradungense* (Satake) Praj. & J.Parn.)	Naiki, A. T4557 (TNS)	Thailand	TD5005	LC485785	LC486055	LC485639	-	LC484490
E. ubonense f. *kradungense* (Satake) Praj. & J.Parn.	Prajaksood, A. AP410 (KKU)	Thailand	AP410	LC485786	LC486056	LC485640	-	-

E. alpestre Hook.f. & Thomson ex Körn.	Tanaka, N. CED084 (TNS)	Japan: Ibaraki	CED084	LC485787	LC486057	LC485641	-	-
E. buergerianum Körn.	TCMK 493 (K)	n/a		-	KC123360	-	-	-
E. hondoense Satake (= *E. taquetii* Lecomte)	Tsugaru, S. & Sawada, T. 35678 (TNS:01156461)	Japan	TD5045	LC485788	LC486058	-	-	-
E. miquelianum Körn.	Hosokawa, K. H-6 (TNS:01137036)	Japan	TD5047	-	LC486059	-	-	-
E. miquelianum Körn.	Ito, Y. YI2347 (TNS)	Japan	YI2347	LC485789	LC486060	LC485642	-	LC484491
E. sikokianum Maxim. (= *E. miquelianum* Körn.)	Enju, C. CED077 (TNS)	Japan: Aichi	CED077	LC485790	LC486061	-	-	-
E. taquetii Lecomte	Yashiro et al. (TNS:721999)	Japan	TD5016	-	LC486062	-	-	-
E. taquetii Lecomte	Tanaka, N. TD4891 (TNS)	Japan	TD4891	LC485791	LC486063	LC485643	LC484957	LC484492
E. teucszii Lecomte	Goyder, D.J. 8364 (K)	Angola	TD5172	LC485792	LC486064	-	-	-
E. thailandicum Moldenke	Prajaksood, A. AP386 (KKU)	Thailand	AP386	-	LC486065	-	-	-
E. thailandicum Moldenke	Prajaksood, A. AP353 (KKU)	Thailand	AP353	-	LC486066	-	-	-
E. thailandicum Moldenke	Souladeth, P. PS364 (FOF)	Laos	PS364	-	LC486067	-	-	-
E. thailandicum Moldenke	Souladeth, P. PS452 (FOF)	Laos	PS452	LC485793	LC486068	LC485644	-	-
E. tingilomum S.M.Phillips & Mesterházy	Mesterhàzy, A. MASIE300 (BP)	Sierra Leone	TD5456	-	LC486069	-	-	-
E. tingilomum S.M.Phillips & Mesterházy	Mesterhàzy, A. MAIVO143 (BP)	Ivory Coast	TD5459	LC485794	LC486070	LC485645	LC484958	-
E. tonkinense Ruhland (= *E. fluviatile* Trimen)	Gale, S.W. SG1371 (KFBG)	China: Hong Kong	SG1371	LC485795	LC486071	LC485646	LC484959	LC484493
E. tonkinense Ruhland (= *E. fluviatile* Trimen)	Gale, S.W. SG1374 (KFBG)	China: Hong Kong	SG1374	LC485796	LC486072	LC485647	-	LC484494
E. sp. Myanmar (cf. *E. truncatum* Buch.-Ham. ex Mart.)	Tanaka, N. NT84 (TNS)	Myanmar	NT84	LC485797	LC486073	LC485648	-	LC484495
E. truncatum Buch.-Ham. ex Mart.	Prajaksood, A. AP454 (KKU)	Thailand	AP454	LC485798	LC486074	-	-	-
E. truncatum Buch.-Ham. ex Mart.	Mbago, F.M. et al. 1872 (K)	Tanzania	TD5152	LC485799	LC486075	LC485649	-	-
E. truncatum Buch.-Ham. ex	Gale, S.W. SG1373	China:	SG1373	LC485800	LC486076	-	-	-

Mart.	(KFBG)	Hong Kong							
E. truncatum Buch.-Ham. ex Mart.	Ito, Y. YI2264 (TNS)	Japan: Okinawa	YI2264	LC485801	LC486077	LC485650	-	LC484496	
E. truncatum Buch.-Ham. ex Mart.	Ito, Y. YI2300 (TNS)	Malaysia	YI2300	LC485802	LC486078	LC485651	-	LC484497	
E. sp. Cambodia	Fuse, S. 6360 (TNS)	Cambodia	TD5004	LC485803	LC486079	-	-	-	
E. sp. Cambodia	Yahara, T. 4565 (TNS)	Cambodia	TD4999	LC485804	LC486080	LC485652	-	LC484498	
E. sp. Laos	Gale, S.W. SG1458 (HNL/TNS)	Laos	SG1458	LC485805	LC486081	LC485653	-	LC484499	
E. ubonense Lecomte	Cho et al. CB-1729 (HHU)	Cambodia	CB1729	LC485806	LC486082	LC485654	-	LC484500	
E. ubonense Lecomte	Cho et al. CB-2735 (HHU)	Cambodia	CB2735	-	LC486083	LC485655	-	-	
E. varium Kimp.	Bidgood, S. et al. 4556 (K)	Tanzania	TD5153	LC485807	LC486084	-	-	-	
E. xeranthemum Mart.	Prajaksood, A. AP382 (KKU)	Thailand	AP382	LC485808	LC486085	LC485656	-	-	
E. zollingerianum Körn.	Gale, S.W. et al. HNL-KFBG 878 (HNL)	Laos	HNL0878	LC485809	LC486086	LC485657	-	-	
